# Health conditions in spousal caregivers of people with dementia and their relationships with stress, caregiving experiences, and social networks: longitudinal findings from the IDEAL programme

**DOI:** 10.1186/s12877-024-04707-w

**Published:** 2024-02-19

**Authors:** Serena Sabatini, Anthony Martyr, Anna Hunt, Laura D. Gamble, Fiona E. Matthews, Jeanette M. Thom, Roy W. Jones, Louise Allan, Martin Knapp, Catherine Quinn, Christina Victor, Claire Pentecost, Jennifer M. Rusted, Robin G. Morris, Linda Clare

**Affiliations:** 1 Institute of Mental Health, School of Medicine, University of Nottingham, Nottingham, UK; 2 University of Exeter Medical School, University of Exeter, Exeter, UK; 3 Population Health Sciences Institute, Newcastle University, Newcastle upon Tyne, UK; 4 Institute for Clinical and Applied Health Research, Hull York Medical School, University of Hull, Hull, UK; 5 Faculty of Medicine and Health, The University of Sydney, Sydney, Australia; 6 Research Institute for the Care of Older People (RICE), Bath, UK; 7 NIHR Applied Research Collaboration South-West Peninsula, Exeter, UK; 8 London School of Economics and Political Science, London, UK; 9 Centre for Applied Dementia Studies, Bradford University, Bradford, UK; 10 Wolfson Centre for Applied Health Research, Bradford, UK; 11 College of Health, Medicine and Life Sciences, Department of Health Sciences, Brunel University London, London, UK; 12 School of Psychology, University of Sussex, Brighton, UK; 13 Institute of Psychiatry, Psychology and Neuroscience, King’s College London, London, UK; 14 Centre for Research in Ageing and Cognitive Health, University of Exeter Medical School, Exeter, UK

**Keywords:** Physical health, Health inequalities, Co-morbidity, Dementia carers, Alzheimer’s disease

## Abstract

**Objectives:**

Longitudinal evidence documenting health conditions in spousal caregivers of people with dementia and whether these influence caregivers’ outcomes is scarce. This study explores type and number of health conditions over two years in caregivers of people with dementia and subgroups based on age, sex, education, hours of care, informant-rated functional ability, neuropsychiatric symptoms, cognition of the person with dementia, and length of diagnosis in the person with dementia. It also explores whether over time the number of health conditions is associated with caregivers’ stress, positive experiences of caregiving, and social networks

**Methods:**

Longitudinal data from the IDEAL (Improving the experience of Dementia and Enhancing Active Life) cohort were used. Participants comprised spousal caregivers (*n* = 977) of people with dementia. Self-reported health conditions using the Charlson Comorbidity Index, stress, positive experiences of caregiving, and social network were assessed over two years. Mixed effect models were used

**Results:**

On average participants had 1.5 health conditions at baseline; increasing to 2.1 conditions over two years. More health conditions were reported by caregivers who were older, had no formal education, provided 10 + hours of care per day, and/or cared for a person with more neuropsychiatric symptoms at baseline. More baseline health conditions were associated with greater stress at baseline but not with stress over time. Over two years, when caregivers’ health conditions increased, their stress increased whereas their social network diminished

**Discussion:**

Findings highlight that most caregivers have their own health problems which require management to avoid increased stress and shrinking of social networks

**Supplementary Information:**

The online version contains supplementary material available at 10.1186/s12877-024-04707-w.

## Background

There are 55 million people with dementia worldwide [1]. As cognitive and functional difficulties increase with the progression of dementia, the amount of care needed by people with dementia increases over time. Just under two-thirds (60%) of people with dementia live in the community and are supported informally by family and friends [2]. There are more than 670,000 informal caregivers of people with dementia in the UK [3, 4] and more than 11 million in the USA [5]. Typically, the spouse or co-habiting partner of the person with dementia assumes the primary caregiving role [1, 6]. Spouses of people with dementia tend to be over 65 and often have their own health conditions to manage [7–13]. Indeed, estimations suggest that just over half of people aged over 65 in the UK and close to 80% in USA have at least one health condition [14–16].

Compared to their peers of similar age, caregivers of people with dementia may have poorer physical and/or mental health and may be at risk of future health decline for several reasons [17–21]. Firstly, caregivers dedicate a lot of their time and energy to the care of the person with dementia and, due to this, they may neglect their own care needs. Previous evidence suggests that, whilst caregivers believe that engaging in health-enhancing behaviors such as doing physical exercise and eating a balanced diet are important for their health, they also generally perceive their needs as less of a priority than the needs of the person with dementia [12, 22, 23]. Secondly, caregiving for a person with dementia, especially where care needs are high, can be stressful and accumulated stress over time can lead to poor mental and physical health [24–29]. For example, chronic stress in caregivers of people with dementia is associated with metabolic syndrome [18]; a potentially treatable cluster of risk factors specific for coronary heart disease, diabetes, and stroke. Thirdly, caregivers of people with dementia often experience social isolation and loneliness which can have a negative impact on their mental health [30–33]. Finally, caregiving for someone with dementia can be physically and mentally exhausting and may have a direct negative impact on the health of the caregiver [34]. This negative impact is not consistently reported, however, and some research has suggested that caregivers are heathier than non-caregivers [35]. This finding has been termed the “healthy caregivers hypothesis”; namely that people who are healthy are more likely to accept the caregiving role than people who are less healthy [35].

Caregivers of people with dementia report poorer health-related quality of life than non-caregivers [36, 37], however, there is limited longitudinal evidence regarding the specific type and number of health conditions that caregivers of people with dementia have, as well as how these change over time. By gaining a better understanding of the health conditions of caregivers of people with dementia across different age groups, sexes, educational attainment backgrounds, hours of care provided per day, and time spent caregiving, healthcare providers can gain valuable insights into the healthcare needs of caregivers of people with dementia [38].

Cross-sectional evidence has shown that caregiving while being in poor physical health may be particularly stressful and burdensome [39, 40]. When caregivers have high levels of stress, it can have negative impacts on their quality of life and psychological well-being, as well as the quality of life and psychological well-being of the person with dementia for whom they are providing care [41–43]. Even though caregivers of people with dementia can have negative experiences of caregiving and find their role stressful, positive experiences of caregiving can co-exist with caregiving-related stress [44]. Positive experiences of caregiving do not simply equal absence of stress but can include for example feelings of competence and self-efficacy related to the caregiving role [44]. Whether poor physical health in caregivers reduces positive experiences of caregiving, in addition to increasing stress, is unknown. Investigating this would be important as positive experiences of caregiving have an effect on caregivers’ well-being and quality of life [44].

Moreover, as older people in better health have a larger social network than older people with poorer health, this likely extends to caregivers [45–47]. In addition, a recent scoping review found that undertaking the caregiving role may lead to a rapid decrease in the social network size of caregivers of people with dementia [48], though social networks may increase over time through connection with other caregivers [48]. This may however be less likely among caregivers with one or more health conditions as managing their own health conditions, in addition to providing help and support to the person with dementia, may leave little time for increasing networks or interacting with existing networks.

The ‘Model of Carer Stress and Burden’ [49] suggests that dementia severity has an impact on caregivers by increasing their workload and burden. Those caregivers who have fewer resources, including poorer health, may find it harder to cope with their role. This may lead to negative psychosocial outcomes in the caregiver including higher caregiving-related stress, more negative and fewer positive experiences of caregiving, and shrinking of their social network due to resources and time being allocated to one’s condition and the care of the person with dementia. Living with dementia has previously been proposed as a shrinking world [50], it is possible that this reduction in opportunities to socialize and explore outdoor activities may affect caregivers in a similar way to how it affects people with dementia.

As there is limited longitudinal research investigating whether caregivers have a greater number of health conditions, they are at greater risk of more stress, fewer positive experiences of caregiving and a smaller social network, it is crucial to develop a better understanding of the health profile of caregivers of people with dementia. It is also important to investigate whether their health status is associated with increased levels of stress, fewer positive experiences of caregiving, and a reduction in social network over time. This is essential in providing informed and effective health and social care services that meet the needs of caregivers of people with dementia.

This study aims to (1) describe the type and number of health conditions at baseline, 12-month, and 24-month follow-up in a large sample of spousal caregivers of people with dementia living in Great Britain and among subgroups based on age, sex, and educational attainment; (2) investigate whether the number of health conditions changes over two years in the overall study sample, among subgroups based on age, sex, education, hours of care per day, time since diagnosis, and based on levels of informant-rated functional ability, neuropsychiatric symptoms, and cognition of the person with dementia; (3) investigate whether over two years those with more health conditions at baseline and over time report higher levels of stress, fewer positive experiences of caregiving, and a smaller social network at baseline and over time.

## Methods

This study used data for spousal caregivers of people with dementia collected in the first three timepoints (baseline: 2014-16; 12-month follow-up: 2015-17; and 24-month follow-up: 2016-18) of the Improving the experience of Dementia and Enhancing Active Life (IDEAL) programme [51]. For each person with dementia in IDEAL, a caregiver, where available, was invited to take part. In IDEAL a caregiver was defined as the primary person who provides practical or emotional unpaid support, usually a family member [41]. People with dementia were recruited through 29 National Health Service sites, and via the online Join Dementia Research portal. Inclusion criteria for people with dementia were at baseline a clinical diagnosis of any type of dementia, having mild-to-moderate dementia (as indicated by a score of ≥ 15 in the Mini-Mental State Examination [52]), and living in the community [51]. There were no specific inclusion criteria for caregivers other than being willing to take part. Further information about the IDEAL study is reported in the published protocol [51]. Analyses were conducted using version 7 of the datasets. At baseline, 1537 people with dementia and 1277 caregivers participated in the IDEAL study. Out of the 1277 caregivers that took part in IDEAL, the present study analyses are based on a subgroup of caregivers who were spouses or partners of the person with dementia, who continued being the primary caregiver from one timepoint to the next, and who provided data on their own health conditions at least once during the three timepoints. The number of caregivers meeting these criteria was 977 at baseline, 802 at 12-month follow-up, and 604 at 24-month follow-up.

The IDEAL study was approved by the Wales 5 Research Ethics Committee (reference: 13/WA/0405) and the Ethics Committee of the School of Psychology, Bangor University (reference: 2014–11,684), and is registered with the UK Clinical Research Network (registration number: 16,593).

### Procedure and measures

For the purpose of present study analyses, the following measures were selected from the wider IDEAL datasets. The following measures were administered at all three timepoints.

### Caregivers self-rated measures

**Health conditions** were assessed with the Charlson Comorbidity Index (CCI) [53, 54]. Health conditions were included in the CCI based on their link with mortality [54]. The CCI was expanded in 2008 by the inclusion of four additional conditions [53]. Example health conditions included in the CCI are cerebrovascular disease, dementia, diabetes, and cancer within the last five years and if present whether it had metastasized. Some conditions are considered superordinate and contain some subtypes; for instance, cerebrovascular disease is treated as superordinate with stroke, cerebrovascular accident, and transient ischemic attack as specific subordinate conditions. Caregivers reported whether they had any of the 23 superordinate conditions, including leukemia and lymphoma. A count of the conditions was used to enumerate health conditions.

**Stress** was assessed with the 15-item Relative Stress Scale [26]. A sample question is *“Do you ever feel that you need a break?”* Participants responded on a Likert scale ranging from 1 = “never” to 5 = “always.” Higher scores (possible range: 0–60) indicate greater stress.

**Positive experiences of caregiving** were assessed with the 9-item Positive Aspects of Caregiving scale [55]. A sample question includes: *“Providing help to my relative/friend has made me feel more useful.”* Participants responded on a Likert scale ranging from 1 = “disagree a lot” to 5 = “agree a lot.” Higher scores (possible range: 9–45) indicate more positive experiences of caregiving.

**Social network** was assessed with the 6-item Lubben Social Network Scale [56]. Sample question is *“How many relatives do you see or hear from at least once a month?”* (response options: 0 = None; 1 = One; 2 = Two; 3 = Three or four; 4 = Five to eight; 5 = Nine or more). Higher scores (possible range: 0–30) indicate larger social networks.

**Hours of Care per Day** was assessed with a categorical variable: less than one hour of care per day; one to ten hours of care per day; more than ten hours of care per day. This variable was treated as ordinal in the study analyses.

**Personal characteristics** comprised age, sex, and education (grouped as: no educational qualifications, school leaving certificate at age 16, school leaving certificate at age 18, university).

### Informant-rated measures

**Functional ability** was assessed with a slightly-modified 11-item version of the Functional Activities Questionnaire [57] containing an additional question concerning telephone use [58, 59]. Higher scores (range: 0–33) indicate poorer functional ability.

**Neuropsychiatric symptoms** were assessed with the informant-rated Neuropsychiatric Inventory Questionnaire [60, 61]. This measure comprises 12 symptoms about changes in sleep problems, apathy, delusion, depression, anxiety, euphoria, agitation, appetite, hallucinations, disinhibition, irritability, and aberrant motor behaviour. The total score (range: 0–12) indicates how many symptoms are present in the person with dementia.

### People with dementia measures

**Cognition** of the person with dementia was assessed with the Addenbrooke’s Cognitive Examination-III [62]. This is a widely used cognitive screening measure that includes five subscale measuring attention, verbal fluency, language, memory, and visuospatial aspects of cognition. Only the total score was used in the analysis. Higher scores (range: 0-100) indicate better cognition.

**Living situation** was a categorical variable comprising three groups: living alone; live with spouse/partner; live with others [63].

**Time since diagnosis** was a categorical variable comprising three groups: Less than one year; between one and two years; three or more years. This was used as a proxy for how long caregivers have spent caregiving [64].

### Analyses

Descriptive statistics for study variables at baseline, 12-month, and 24-month follow-ups were reported. Number and proportion of caregivers with each of the investigated health conditions at baseline, 12-month, and 24-month follow-ups were reported.

A nested case-control approach was used to determine whether number of health conditions was associated with dropout. This involved logistic regression models to determine whether dropout was associated with number of health conditions at the previous timepoint. Estimates were combined using *metan* in Stata [65].

### Health conditions over time

Mixed effect models with a Poisson distribution were used to investigate number of health conditions at baseline and the trajectory of change in number of health conditions over two years. The model estimated incidence rate ratios (IRR) associated with the intercept and a slope, with random effects to account for variation across individuals. Models comprised an unadjusted model, a partially adjusted model (adjusted for age, sex, education, hours of care per day, and time since diagnosis), and a fully adjusted model (adjusted for age, sex, education, hours of care per day, time since diagnosis, informant-rated functional ability, neuropsychiatric symptoms, and cognition of the person with dementia).

### Health conditions as a function of risk factors

Associations of age, sex, education, hours of care per day, time since diagnosis, informant-rated functional ability, neuropsychiatric symptoms, and cognition of the person with dementia with the number of health conditions in caregivers were explored at baseline and over two years. An unadjusted model, a partially adjusted model, and a fully adjusted model were estimated, as described above. The fully adjusted model was also estimated with hours of care per day, informant-rated functional ability, neuropsychiatric symptoms, and cognition of the person with dementia as time-varying predictors of number of health conditions at baseline and over time.

### Health conditions as predictors for caregiver outcomes

Linear mixed effect models were used to investigate whether number of health conditions at baseline were associated with stress, positive experiences of caregiving, and social network, at baseline and over time. An unadjusted model, a partially adjusted model, and a fully adjusted model were estimated, as described above. Linear mixed effects models were also estimated for number of health conditions and covariates as time-varying predictors of stress, positive experiences of caregiving, and social network at baseline and over time. Within and between-person effects were reported.

Analyses were conducted using Stata version 17 [66].

## Results

### Descriptive statistics

At baseline, the mean age of caregivers was 72.29 and approximately two-thirds were women. In this sample, 42.2% of caregivers provided more than ten hours of care per day, 36.9% provided between one to ten hours of care per day and 20.0% provided less than one hour of caregiving per day. Demographic characteristics of the sample at follow-ups were similar to baseline. Descriptive statistics for all study variables at all timepoints are reported in Table 1.

**Table 1:** Descriptive statistics for study variables at baseline, 12-month, and 24-month follow-ups

	Baseline (*N* = 977)	12-month follow-up (*N* = 802)	24-month follow-up (*N* = 604)
**Caregiver variables**			
**Demographic variables**			
Age in years, M (SD; range)	72.29 (8.19; 41–92)	73.21 (7.95; 42–93)	73.76 (7.85; 32–94)
Age group, n (%)			
< 65 years	149 (15.2)	106 (13.2)	63 (10.4)
65–69	191 (19.5)	137 (17.1)	104 (17.2)
70–74	241 (24.6)	207 (25.8)	164 (27.1)
75–79	207 (21.3)	174 (21.7)	125 (20.8)
≥ 80	189 (19.4)	178 (22.2)	148 (24.5)
Ethnicity, n (%)			
White British	942 (96.4)	774 (96.5)	582 (96.4)
White other	28 (2.9)	22 (2.8)	18 (3.0)
Other	7 (0.7)	6 (0.7)	4 (0.6)
Sex, n (%)			
Women	655 (67.0)	533 (66.5)	395 (65.5)
Men	322 (33.0)	269 (33.5)	209 (34.5)
Education, n (%)			
No qualifications	237 (24.3)	190 (23.7)	138 (22.9)
School leaving certificate age 16	225 (23.1)	187 (23.4)	140 (23.3)
School leaving certificate age 18	281 (28.9)	227 (28.4)	170 (28.2)
University level education	231 (23.7)	196 (24.5)	154 (25.6)
Missing, n	3	2	2
Hours of care per day, n (%)			
Less than one hour of care per day	195 (20.0)	122 (15.7)	75 (12.5)
One to ten hours of care per day	361 (36.9)	277 (35.6)	221 (36.9)
More than ten hours of care per day	412 (42.2)	380 (48.8)	303 (50.6)
Missing, n	9	23	5
Health conditions, M (SD)	1.46 (1.42)	1.85 (1.65)	2.07 (1.85)
No health conditions, n (%)	261 (26.8)	159 (19.8)	107 (17.7)
One health condition, n (%)	315 (45.5)	208 (34.2)	126 (27.3)
Two health conditions, n (%)	202 (29.1)	194 (31.9)	156 (33.8)
Three health conditions, n (%)	99 (14.3)	110 (18.1)	93 (20.2)
Four health conditions, n (%)	41 (5.9)	42 (6.9)	42 (9.1)
Five health conditions, n (%)	19 (2.7)	26 (4.3)	20 (4.3)
Six health conditions, n (%)	8 (1.2)	10 (1.6)	10 (2.2)
Seven health conditions, n (%)	5 (0.7)	9 (1.5)	6 (1.3)
Eight health conditions, n (%)	3 (0.4)	7 (1.2)	5 (1.1)
Nine health conditions, n (%)	1 (0.1)	1 (0.2)	1 (0.2)
Ten health conditions, n (%)	0 (0)	1 (0.2)	2 (0.4)
Missing, n	23	35	36
Stress, M (SD)	19.38 (9.75)	22.15 (10.11)	23.29 (10.13)
Missing, n	43	54	42
Positive aspects of caregiving, M (SD)	27.94 (7.42)	27.72 (7.75)	27.73 (7.82)
Missing, n	20	40	31
Social network, M (SD)	17.70 (5.43)	17.34 (5.30)	17.10 (5.34)
Missing, n	24	39	28
**Informant/caregiver-rated variables**			
Functional ability, M (SD)	17.56 (8.55)	20.67 (8.68)	22.62 (8.70)
Missing, n	62	36	21
Neuropsychiatric symptoms, M (SD)	3.42 (2.43)	3.77 (2.53)	4.13 (2.51)
Missing, n	202	18	23
**Person with dementia variables**			
Cognition, M (SD)	69.56 (13.92)	65.97 (16.44)	64.15 (18.63)
Missing, n	183	71	101
Time since diagnosis, n (%)			
Less than one year	479 (49.0)		
Between one and two years	298 (30.5)		
Three or more years	134 (13.7)		
Missing, n	66		

At baseline, 73.2% of caregivers reported having at least one of the included health conditions. This proportion increased to 80.2% and 82.3% at 12-month and 24-months follow-up, respectively. At baseline, the most health conditions caregivers had were nine, and this increased to ten at 12-month and 24-month follow-ups. Amongst the most frequently reported health conditions by caregivers at baseline, hypertension/high blood pressure was reported by about one third and inflammation affecting the joints was reported by a quarter. Cancer within the last five years was reported by 11.7% of caregivers. Chronic bad chest, depression, and diabetes controlled with insulin or equivalent were reported by between about 8% and 11% of caregivers. See Table 2 for the details of each health condition.

**Table 2:** Number and percentage of participants with each health condition at baseline, 12-month, and 24-month follow-ups

	Baseline (*N* = 954)	12-month follow-up (*N* = 767)	24-month follow-up (*N* = 568)
**Health conditions**			
	Yes, N (%)		
Myocardial infarction (history of heart attacks)	53 (5.6)	53 (6.9)	44 (7.7)
Congestive heart failure	10 (1.0)	15 (2.0)	20 (3.5)
Hypertension/high blood pressure	359 (37.6)	349 (45.5)	274 (48.2)
Diagnosed depression	77 (8.1)	81 (10.6)	67 (11.8)
Peripheral vascular disease	47 (4.9)	66 (8.6)	56 (9.9)
Aortic aneurysm	7 (0.7)	10 (1.3)	9 (1.6)
Poor circulation	27 (2.8)	40 (5.2)	34 (6.0)
Cerebrovascular disease	50 (5.2)	55 (7.2)	43 (7.6)
Stroke	18 (1.9)	25 (3.3)	20 (3.5)
Cerebrovascular accident	1 (0.1)	0 (0)	2 (0.4)
Transient Ischemic attack	25 (2.6)	30 (3.9)	24 (4.2)
Dementia	49 (5.1)	44 (5.7)	30 (5.3)
Chronic bad chest	103 (10.8)	115 (15.0)	100 (17.6)
Asthma	65 (6.8)	74 (9.6)	67 (11.8)
Chronic obstructive pulmonary disease	28 (2.9)	35 (4.6)	34 (6.0)
Chronic bronchitis	8 (0.8)	8 (1.0)	7 (1.2)
Emphysema	4 (0.4)	8 (1.0)	7 (1.2)
Inflammation affecting the joints	246 (25.8)	276 (36.0)	230 (40.5)
Lupus	3 (0.3)	1 (0.1)	1 (0.2)
Rheumatoid arthritis	91 (9.5)	119 (15.5)	106 (18.7)
Connective tissue disease	5 (0.5)	5 (0.7)	6 (1.1)
Vasculitis	3 (0.3)	2 (0.3)	2 (0.4)
Peptic/stomach ulcer disease	27 (2.8)	29 (3.8)	25 (4.4)
Skin ulcer	9 (0.9)	11 (1.4)	11 (1.9)
Bed sores	0 (0)	0 (0)	0 (0)
Repeated cellulitis	7 (0.7)	6 (0.8)	6 (1.1)
Diabetes controlled with insulin or equivalent	75 (7.9)	75 (9.8)	55 (9.7)
Diabetes with end organ damage	32 (3.4)	34 (4.4)	30 (5.3)
Damage to the retina	9 (0.9)	8 (1.0)	8 (1.4)
Nerve damage	4 (0.4)	4 (0.5)	6 (1.1)
Kidney damage	5 (0.5)	9 (1.2)	9 (1.6)
Brittle diabetes	0 (0)	0 (0)	1 (0.2)
Moderate or severe chronic kidney disease	15 (1.6)	18 (2.3)	18 (3.2)
Hemiplegia	1 (0.1)	0 (0)	1 (0.2)
Cancer within the last five years	112 (11.7)	118 (15.4)	101 (17.8)
Breast cancer	14 (1.5)	17 (2.2)	16 (2.8)
Colon cancer	5 (0.5)	3 (0.4)	4 (0.7)
Prostate cancer	14 (1.5)	19 (2.5)	16 (2.8)
Lung cancer	1 (0.1)	1 (0.1)	2 (0.4)
Skin cancer	22 (2.3)	30 (3.9)	27 (4.8)
Blood cancer/lymphoma	4 (0.4)	7 (0.9)	9 (1.6)
Acute or chronic leukemia	3 (0.3)	6 (0.8)	6 (1.1)
Cancer within the past five years that has metastasized	42 (4.4)	36 (4.7)	26 (4.6)
Mild liver disease	4 (0.4)	5 (0.7)	5 (0.9)
Hepatitis B	1 (0.1)	1 (0.1)	1 (0.2)
Hepatitis C	0 (0)	1 (0.1)	1 (0.2)
Cirrhosis	1 (0.1)	1 (0.1)	0 (0)
Liver disease (moderate to severe)	6 (0.6)	5 (0.7)	2 (0.4)
Chronic jaundice	2 (0.2)	2 (0.3)	1 (0.2)
Liver failure	1 (0.1)	1 (0.1)	0 (0)
Liver transplant	0 (0)	0 (0)	0 (0)
AIDS or HIV	0 (0)	0 (0)	0 (0)
Taking warfarin	69 (7.2)	55 (7.2)	44 (7.7)

In the present study we were interested in the count of health conditions. However, conditions that are typically weighted as having a higher impact on health and the cost of care are hemiplegia, moderate or severe chronic kidney disease, diabetes with end of organ damage, any cancer, skin ulcers/cellulitis, moderate or severe live disease, metastatic cancer, and AIDS or HIV. In the weighted CCI total score all these conditions receive a weight of two except for moderate or severe liver disease which receives a weight of three, and metastatic cancer and AIDS or HIV which receive a weight of six. The remaining conditions, and taking warfarin, receive a weight of one [53]

### Number of health conditions over time in the overall study sample

At baseline the mean number of health condition was 1.46 (Table 1). The number of health conditions increased over the study period in the unadjusted mixed effects model (IRR = 1.20; 95% CI: 1.15, 1.25), as well as in the partially adjusted model (IRR = 1.19; 95% CI: 1.14, 1.23) and in the fully adjusted model with variables as time-varying predictors (IRR = 1.20; 95% CI: 1.13; 1.28).

### Baseline and change in number of health conditions as a function of risk factors

At baseline, caregivers aged under 70 years were more likely to have fewer health conditions compared to caregivers aged 70–74 years (Table 3; Supplementary Table 1). At baseline, the number of health conditions did not differ between men and women (Table 3; Supplementary Table 2). At baseline, those with university level education were more likely to have fewer health conditions than those with no educational qualifications (Table 3; Supplementary Table 3). There was no association between age, sex, or education with number of health conditions over time.

**Table 3:** Associations of age, sex, and education with health conditions at baseline and over time

	Unadjusted model		Partially adjusted model		Fully adjusted model	
	Mean intercept (IRR, 95% CI)	Mean slope (IRR, 95% CI)	Mean intercept (IRR, 95% CI)	Mean slope (IRR, 95% CI)	Mean intercept (IRR, 95% CI)	Mean slope (IRR, 95% CI)
**Caregivers variables**						
Age group (reference: 70–74)						
<65 years	0.71 (0.56; 0.89)	0.98 (0.85; 1.13)	0.70 (0.56; 0.89)	0.98 (0.85; 1.13)	0.68 (0.53; 0.87)	0.98 (0.84; 1.15)
65–69	0.81 (0.66; 1.00)	0.98 (0.87; 1.11)	0.81 (0.65; 1.00)	0.99 (0.87; 1.12)	0.76 (0.61; 0.95)	0.99 (0.87; 1.14)
75–79	0.98 (0.81; 1.20)	1.01 (0.90; 1.13)	0.94 (0.77; 1.16)	0.99 (0.88; 1.12)	0.97 (0.78; 1.20)	0.99 (0.87; 1.13)
≥80	0.93 (0.76; 1.14)	1.07 (0.84; 1.22)	0.89 (0.72; 1.10)	1.09 (0.96; 1.25)	0.97 (0.77; 1.22)	1.10 (0.95; 1.27)
Sex (reference: female)						
Male	1.01 (0.87; 1.17)	0.99 (0.91; 1.08)	1.00 (0.86; 1.17)	0.97 (0.88; 1.06)	1.11 (0.94; 1.32)	0.97 (0.87; 1.07)
Education						
No qualification (Reference category)						
School leaving certificate age 16	0.90 (0.74; 1.09)	0.98 (0.88; 1.10)	0.96 (0.78; 1.17)	0.98 (0.87; 1.10)	1.01 (0.81; 1.25)	0.99 (0.87; 1.12)
School leaving certificate age 18	0.86 (0.72; 1.04)	0.98 (0.88; 1.10)	0.87 (0.72; 1.06)	0.98 (0.87;1.10)	0.89 (0.73; 1.09)	0.98 (0.87; 1.12)
University level education	0.71 (0.58; 0.87)	0.99 (0.88; 1.12)	0.72 (0.58; 0.89)	0.99 (0.87; 1.12)	0.73 (0.59; 0.91)	0.99 (0.86; 1.13)
Hours of care per day						
Less than one hour of care per day	0.76 (0.62; 0.92)	1.02 (0.91; 1.14)	0.78 (0.64; 0.95)	1.03 (0.91; 1.16)	0.78 (0.62; 0.99)	1.06 (0.92; 1.23)
One to ten hours of care per day	0.89 (0.76; 1.04)	1.03 (0.94; 1.13)	0.93 (0.79; 1.09)	1.04 (0.94; 1.15)	0.91 (0.76; 1.08)	1.05 (0.95;1.18)
More than ten hours of care per day (Reference category)						
**Informant/caregiver-rated variables**						
Functional ability	1.01 (0.93; 1.10)	1.01 (0.96; 1.07)			0.93 (0.82; 1.05)	1.02 (0.94; 1.10)
Neuropsychiatric symptoms	1.04 (1.01; 1.07)	1.00 (0.99; 1.02)			1.06 (1.02; 1.10)	1.00 (0.98; 1.02)
**People with dementia variables**						
Cognition	1.04 (0.99; 1.09)	0.99 (0.96; 1.02)			1.08 (1.01; 1.15)	0.99 (0.95; 1.03)
Time since diagnosis						
Less than one year (Reference category)						
Between one and two years	1.06 (0.91; 1.24)	1.02 (0.91; 1.14)	1.03 (0.88; 1.21)	1.01 (0.92; 1.12)	1.05 (0.89; 1.24)	1.01 (0.91; 1.12)
Three or more years	1.11 (0.91; 1.37)	1.03 (0.94; 1.13)	1.07 (0.87; 1.32)	0.99 (0.86; 1.13)	1.09 (0.87; 1.36)	0.97 (0.84; 1.12)

Partially adjusted model: age, sex, education, time since diagnosis, baseline hours of care per day. Fully adjusted model: age, sex, education, time since diagnosis, baseline hours of care per day, baseline informant-rated functional ability, neuropsychiatric symptoms, cognition of the person with dementia. Scores for cognition of the person with dementia and informant-rated functional ability have been divided by 10 due to narrow confidence intervals. IRR: incidence rate ratio

In the unadjusted and fully adjusted models, compared to those providing more than ten hours of care per day, those providing less than one hour of care per day had fewer health conditions at baseline, but there was no significant association between hours of care per day and number of health conditions over time (Table 3; Supplementary Table 4).

Both in the unadjusted and fully adjusted models, informant-rated functional ability was not significantly associated with number of health conditions over time in caregivers (Table 3). Both in the unadjusted and fully adjusted models, more neuropsychiatric symptoms were associated with a higher number of health conditions in caregivers at baseline but not with change in number of health conditions over time (Table 3). In the fully adjusted model, but not in the unadjusted model, people with dementia with better cognition was associated with more health conditions in the caregiver (Table 3). Both in the unadjusted and fully adjusted models, time since diagnosis was not associated with number of health conditions in caregivers at baseline nor over time (Table 3; Supplementary Table 5).

Supplementary Table 6 reports results of unadjusted and adjusted models for informant-rated functional ability, neuropsychiatric symptoms, and cognition of the person with dementia as time-varying predictors of number of health conditions over time. Informant-rated functional ability, neuropsychiatric symptoms, and cognition of the person with dementia did not change over time not at the within- nor at the between-person level. Within- and between-person informant-rated functional ability and cognition of the person with dementia were not significant predictors of risk of number of health conditions in the caregiver over time nor in the unadjusted nor in the adjusted model. An increase in between-person (multivariable model IRR = 1.05; 95% CI: 1.02; 1.09), but not in within-person, neuropsychiatric symptoms were associated with increased risk of number of health conditions in the carer over time.

A nested case-control approach was used to determine whether number of health conditions was associated with dropout from the study. This analysis suggests that the number of health conditions was not associated with dropout; combined estimate OR = 0.93; (95% CI: 0.86, 1.01); T1-T2 OR = 0.92 (95% CI: 0.81, 1.04), and T2-T3 OR = 0.94 (95% CI: 0.85, 1.04).

### Number of health conditions at baseline and over time as predictor of baseline and change in stress, positive experiences of caregiving, and social network over two years

**Fig. 1:**
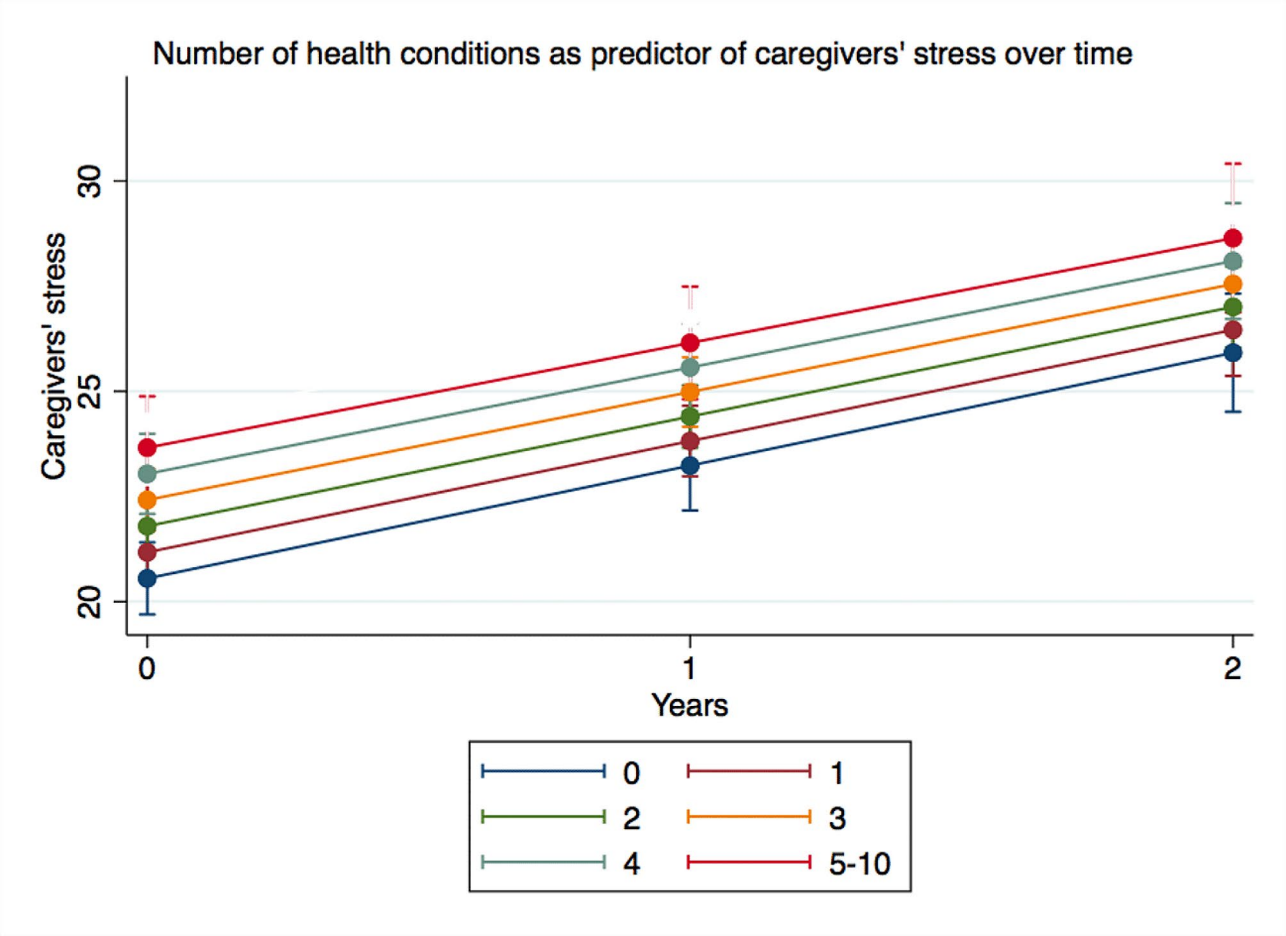
Number of health conditions as predictor of caregivers’ stress over time. Note: numbers from 0 to 5–10 indicate the number of health conditions in caregivers

Longitudinal analysis is presented in Table 4. Caregivers reported relatively low levels of stress at baseline. Stress remained relatively low over the two years, though levels of stress significantly increased over time by nearly three points each year (fully adjusted model mean slope = 2.74; 95% CI: 2.44, 3.05). Having more health conditions was associated with higher levels of stress at baseline (Fig. 1) but not with change in stress over time. Positive experiences of caregiving did not significantly change over time (fully adjusted model mean slope=-0.13; 95% CI: -0.38, 0.11), and this was unrelated to the number of health conditions that caregivers reported at baseline and over time. At baseline, caregivers scored on average 17 out of 30 on the Lubben Social Network Scale; which equates to caregivers seeing approximately three to four different friends/family members per month. Scores on the Lubben Social Network Scale slightly decreased over time (fully adjusted model mean slope= -0.37; 95% CI: -0.53, -0.20), and this was unrelated to the number of health conditions reported by caregivers at baseline and over time.

**Table 4:** Health conditions as predictors of stress, positive aspects of caregiving, and social network over time

	Stress (estimate, 95% CI)	Positive aspects of caregiving (estimate, 95% CI)	Social network (estimate, 95% CI)
Unadjusted model			
Intercept (Health conditions at baseline)	0.47 (0.04; 0.90)	0.40 (0.07; 0.72)	-0.14 (-0.37; 0.09)
Slope (Health conditions x time)	0.13 (-0.08; 0.34)	0.03 (-0.14; 0.20)	-0.05 (-0.17; 0.06)
Partially adjusted model one			
Intercept (Health conditions at baseline)	0.33 (-0.09; 0.36)	0.21 (-0.13; 0.55)	-0.12 (-0.37; 0.13)
Slope (Health conditions x time)	0.16 (-0.05; 0.36)	0.03 (-0.15; 0.21)	-0.06 (-0.18; 0.06)
Fully adjusted model two			
Intercept (Health conditions at baseline)	0.04 (-0.35; 0.43)	0.22 (-0.16; 0.59)	-0.05 (-0.32; 0.22)
Slope (Health conditions x time)	0.12 (-0.11; 0.34)	0.07 (-0.13; 0.26)	-0.09 (-0.21; 0.04)

Partially adjusted model: age, sex, education, hours of care per day, time since diagnosis. Fully adjusted model: age, sex, education, hours of care per day, time since diagnosis, neuropsychiatric symptoms, informant-rated functional ability, cognition of the person with dementia

Supplementary Table 7 reports results for number of health conditions and covariates as time-varying predictors of stress, positive aspects of caregiving, and social network.

In the unadjusted, partially adjusted, and fully adjusted models at within-person level, when number of health conditions increase, stress also increases (fully adjusted model estimate: 1.19; 95% CI: 0.69; 1.69), and social network decreases (fully adjusted model estimate: -0.32; 95% CI: -0.64; -0.00). At the within-person level the association between number of health conditions as time-varying covariate of positive aspects of caregiving was not statistically significant. At the between-person level none of the associations were significant in the fully adjusted models.

## Discussion

This study reported the overall type and number of CCI health conditions in a large sample of spousal caregivers of people with dementia living in Great Britain and examined any associations with age, sex, education, hours of care per day, time since diagnosis in the person with dementia, informant-rated functional ability, neuropsychiatric symptoms, and cognition of the person with dementia. This study also investigated whether the number of CCI health conditions changed over two years and whether over the same time period those with more CCI health conditions reported higher levels of stress, fewer positive experiences of caregiving, and/or a smaller social network.

Overall, consistent with evidence from the UK population, findings suggest that 73.2% of spousal caregivers of people with dementia had at least one CCI health condition to manage, especially those who were older, who had no formal educational qualifications, and/or provided more hours of care per day [14]. More neuropsychiatric symptoms in the person with dementia were also found associated with a higher number of health conditions in the caregiver at baseline. Consistent with evidence from older people in England, the most frequent CCI health conditions reported by caregivers were hypertension, inflammation affecting the joints, and cancer within the previous five years [15]. Number of CCI health conditions in caregivers increased over time from 1.5 health conditions at baseline to two health conditions at 24-month follow-up. Caregivers who had more CCI health conditions reported minimally higher levels of stress at baseline, and they did not report having fewer positive experiences of caregiving at baseline or a smaller social network at baseline than those with fewer CCI health conditions. However, we found that for a given individual over time, when number of health conditions increased, stress also increased and the size of their social network decreased.

The findings are consistent with recent evidence showing that a significant proportion of older people in England have two or more health conditions, with 52.8% and 75.9% of those aged between 65 and 74 and aged 75–84, respectively, reporting multiple health conditions [14, 15]. Among spousal caregivers of people with dementia in the present study, the number of health conditions was lower among those aged under 70, compared to those aged 70–74, which aligns with global evidence for the general population of older adults and caregivers of people with dementia [7–13]. Our finding that caregivers had the same average number of health conditions as those reported for non-caregivers in previous studies [7–13] may be due to a self-selection bias, i.e., caregivers who were less healthy were less likely to take part in IDEAL than caregivers who were healthier. However, empirical evidence comparing the health of caregivers of people with dementia with non-caregivers seems inconclusive. Whereas some studies have suggested caregivers are heathier than non-caregivers and this gave rise to the “healthy caregivers hypothesis” (i.e., people who are healthy are more likely to accept the caregiving role than people who were less healthy) [35], most studies have found that caregivers are less healthy than non-caregivers [20, 21]. For example, a meta-analysis found a small effect that non-caregivers have better health than caregivers; there was however a larger effect for non-caregivers having better health than caregivers of people with dementia [21]. Another meta-analysis, focusing on objective health indicators, such as stress hormones, antibodies, and medication use, found that informal caregivers generally have poorer physical health than non-caregivers [20]. Other studies have suggested that caregivers are at greater risk of poorer health than non-caregivers, primarily due to the effect that increased stress has on metabolic syndrome in caregivers [18].

The findings of the present study suggest that the typical caregiver tends to have one or two major health conditions with the potential for increased risk of mortality of their own to manage. Given that caregivers often face challenges in incorporating health-promoting behaviors into their daily routine and may prioritize the needs of the person with dementia over their own needs [12, 22, 23], healthcare professionals should prioritize monitoring the health of caregivers, particularly those showing signs of heightened stress, even when they are not currently receiving treatment for their health conditions [17, 67]. These findings underscore the importance of recognizing and addressing the healthcare needs of caregivers of people with dementia to ensure that they receive appropriate support.

Despite reporting relatively low levels of stress, caregivers who had more CCI health conditions reported greater stress at baseline. Moreover, caregivers who developed more health conditions over the study period also reported increased stress over time. This pattern of results suggest that having any of the 23 conditions included in the CCI may have a negative impact on caregivers’ stress levels. It is important to note that even relatively low levels of sustained stress, if left unmanaged, can have both a detrimental consequences on the health of caregivers [24, 25] and can negatively impact their quality of life, as well as the quality of life of the person with dementia [41–43]. However, some of the health conditions investigated, such as depression, can be connected with higher stress [68]. Therefore, it is crucial to identify and address specific health conditions, including depression, that may contribute to higher stress levels among spousal caregivers of people with dementia, especially given that the impact of different health conditions on stress and caregiving ability may vary greatly. For example, health conditions such as inflammation affecting the joints, when severe, may inhibit certain caregiving tasks that require manual dexterity or lifting.

Additionally, whereas some health conditions, such as hypertension, are more easily manageable, others, such as inflammation affecting the joints, can limit caregiving tasks even when levels of pain are controlled. On average, having more CCI health conditions at baseline did not appear to be related to having a less positive experience of caregiving or to a concomitant decrease in social network size. Even though at baseline the caregivers in the present study had on average a social network size similar to that of a sample of older non-caregivers living in London [56], caregivers who developed more health conditions over the study period experienced a concomitant decrease in the size of their network. This is noteworthy because a large social network can help to maintain emotional well-being while managing health conditions [32, 69, 70]. Our finding is consistent with the associations proposed in the ‘Model of Carer Stress and Burden’ [49] as our results suggest that caregivers of people living with dementia and who are themselves in poor health have fewer resources to support the person with dementia. Over time, this can lead to negative psychosocial outcomes including higher levels of stress in the caregiver and a shrinking of caregivers’ social networks. Understanding the specific impact that different health conditions can have on the ability of caregivers to carry out caregiving tasks is essential. Further research is needed to better understand the relationship between health conditions and caregiving experiences, as well as to identify effective interventions to support caregivers in managing their health conditions and maintaining their social networks and well-being.

This study has some limitations that need to be acknowledged. Health conditions were self-reported by caregivers and not retrieved from medical records, so it is possible that health conditions may be over- or under-reported [71]. Moreover, this study only assessed the selected health conditions included in the CCI, suggesting it is likely that caregivers in the present sample had more health conditions. Additionally, the study and the analysis focused on the total number of health conditions present and did not use the CCI weighting system that scores them according to severity. However, among the CCI conditions that are weighted towards those with a higher health impact, only 0.6% and 4.3% of caregivers had moderate or severe liver disease and/or metastatic cancer, respectively. Therefore, the proportion of caregivers in the study with these major, potentially life-threatening health conditions, was relatively small. Interestingly, this proportion was similar to the incidence of these conditions found in their care recipients [72]. A longer study duration would be useful as the two-year follow-up may be insufficient to fully understand whether and how co-morbidity and/or multimorbidity changes over time in caregivers of people with dementia, especially those aged 70 or over. Finally, because almost all participants were of white ethnicity, which is consistent with the current demographic of dementia caregivers in Great Britain [73], it was not possible to explore the role of race/ethnicity in the analyses.

Nonetheless, the present study has several strengths. It was based on a large sample of spousal caregivers living in Great Britain; this made it possible to reliably explore health conditions among subgroups of participants based on age, sex, education, hours of care per day, and time since diagnosis. It was also based on longitudinal data which made it possible to extend existing cross-sectional evidence linking health conditions with stress, positive experiences of caregiving, and social network size.

## Conclusions

Spousal caregivers of people with dementia appear to have the same number of health conditions as similarly aged older people. However, those caregivers who are older, have no formal educational qualifications, and/or provide more hours of care per day seem to be at greater risk of having more health conditions. Caregivers of people with dementia who developed more health conditions over time also experienced an increase in their levels of stress and a decrease in the size of their social network. However, they do not seem to perceive a decrease in the number of positive experiences of caregiving. Having to manage one’s own health conditions, in addition to the person with dementia, appears to be stressful for caregivers and may limit the time they can spend with friends and family. Further research is needed to better understand the longer-term health impact of caregiving on spousal caregivers.

### Electronic supplementary material

Below is the link to the electronic supplementary material.

Supplementary Material 1

## Data Availability

IDEAL data were deposited with the UK Data Archive in April 2020. Details of how the data can be accessed can be found here: https://reshare.ukdataservice.ac.uk/854293/.

## Abbreviations

IDEAL: Improving the experience of Dementia and Enhancing Active Life
CCI: Charlson Comorbidity Index
OR: Odds ratio
IRR: Incidence rate ratio
95% CI: 95% confidence interval
