# Supplementary material for: Health conditions in spousal caregivers of people with dementia and their relationships with stress, caregiving experiences, and social networks: longitudinal findings from the IDEAL programme
Source: BMC Geriatr. 2024 Feb 19;24:171. doi: 10.1186/s12877-024-04707-w (PMC10875834; doi:10.1186/s12877-024-04707-w)
Supplement: Supplementary file 1 — Supplementary Material 1 [file 12877_2024_4707_MOESM1_ESM.docx]

**Supplementary Table 1.** Number and percentage of participants with each health condition at baseline according to age subgroups.

|  | <65 years  (N=148) | 65-69  (N=190) | 70-74  (N=235) | 75-79  (N=201) | ≥80  (N=180) |
| --- | --- | --- | --- | --- | --- |
| **Health conditions** | Yes, N (%) |  |  |  |  |
| Myocardial infarction (history of heart attacks) | 3 (2.0) | 6 (3.2) | 17 (7.2) | 10 (5.0) | 17 (9.3) |
| Congestive heart failure | 1 (0.7) | 2 (1.1) | 2 (0.9) | 2 (1.0) | 3 (1.7) |
| Hypertension/high blood pressure | 41 (27.7) | 68 (35.8) | 96 (40.9) | 90 (44.8) | 64 (35.2) |
| Diagnosed depression | 19 (12.8) | 19 (10.0) | 27 (11.5) | 8 (4.0) | 4 (2.2) |
| Peripheral vascular disease | 3 (2.0) | 6 (3.2) | 10 (4.3) | 11 (5.5) | 17 (9.3) |
| Aortic aneurysm | 0 (0) | 0 (0) | 1 (0.4) | 3 (1.5) | 3 (1.7) |
| Poor circulation | 2 (1.4) | 4 (2.1) | 7 (3.0) | 7 (3.5) | 7 (3.9) |
| Cerebrovascular disease | 4 (1.4) | 6 (3.2) | 11 (4.7) | 17 (8.5) | 12 (6.6) |
| Stroke | 2 (1.4) | 0 (0) | 6 (2.6) | 7 (3.5) | 3 (1.7) |
| Cerebrovascular accident | 0 (0) | 0 (0) | 0 (0) | 0 (0) | 1 (0.6) |
| Transient Ischemic attack | 3 (2.0) | 3 (1.6) | 4 (1.7) | 8 (4.0) | 7 (3.9) |
| Dementia | 6 (4.1) | 7 (3.7) | 14 (6.0) | 14 (7.0) | 8 (4.4) |
| Chronic bad chest | 18 (12.2) | 16 (8.4) | 31 (13.2) | 21 (10.5) | 17 (9.3) |
| Asthma | 14 (9.5) | 12 (6.4) | 20 (8.5) | 13 (6.5) | 6 (3.3) |
| Chronic obstructive pulmonary disease | 3 (2.0) | 4 (2.1) | 7 (3.0) | 8 (4.0) | 6 (3.3) |
| Chronic bronchitis | 1 (0.7) | 1 (0.5) | 3 (1.3) | 0 (0) | 3 (1.7) |
| Emphysema | 0 (0) | 0 (0) | 1 (0.4) | 2 (1.0) | 1 (0.6) |
| Inflammation affecting the joints | 31 (21.0) | 57 (30.0) | 63 (26.8) | 51 (25.4) | 44 (24.2) |
| Lupus | 1 (0.7) | 0 (0) | 1 (0.4) | 0 (0) | 1 (0.6) |
| Rheumatoid arthritis | 11 (7.5) | 19 (10.1) | 20 (8.5) | 23 (11.4) | 18 (9.9) |
| Connective tissue disease | 1 (0.7) | 3 (1.6) | 1 (0.4) | 0 (0) | 0 (0) |
| Vasculitis | 0 (0) | 2 (1.1) | 1 (0.4) | 0 (0) | 0 (0) |
| Peptic/stomach ulcer disease | 5 (3.4) | 5 (2.6) | 9 (3.8) | 4 (2.0) | 4 (2.2) |
| Skin ulcer | 1 (0.7) | 1 (0.5) | 3 (1.3) | 1 (0.5) | 3 (1.7) |
| Bed sores | 0 (0) | 0 (0) | 0 (0) | 0 (0) | 0 (0) |
| Repeated cellulitis | 0 (0) | 1 (0.5) | 2 (0.9) | 1 (0.5) | 3 (1.7) |
| Diabetes controlled with insulin or equivalent | 12 (8.1) | 12 (6.3) | 22 (9.4) | 11 (5.5) | 18 (9.9) |
| Diabetes with end organ damage | 3 (2.0) | 3 (1.6) | 8 (3.4) | 11 (5.5) | 7 (3.9) |
| Damage to the retina | 2 (1.4) | 1 (0.5) | 1 (0.4) | 0 (0) | 5 (2.8) |
| Nerve damage | 2 (1.4) | 0 (0) | 1 (0.4) | 1 (0.5) | 0 (0) |
| Kidney damage | 0 (0) | 1 (0.5) | 0 (0) | 2 (1.0) | 2 (1.1) |
| Brittle diabetes | 0 (0) | 0 (0) | 0 (0) | 0 (0) | 0 (0) |
| Moderate or severe chronic kidney disease | 0 (0) | 1 (0.5) | 5 (2.1) | 7 (3.5) | 2 (1.1) |
| Hemiplegia | 0 (0) | 0 (0) | 0 (0) | 1 (0.5) | 0 (0) |
| Cancer within the last five years | 11 (7.4) | 20 (10.5) | 32 (13.6) | 30 (14.9) | 19 (10.4) |
| Breast cancer | 1 (0.7) | 3 (1.6) | 5 (2.1) | 5 (2.5) | 0 (0) |
| Colon cancer | 1 (0.7) | 0 (0) | 2 (0.9) | 0 (0) | 2 (1.1) |
| Prostate cancer | 2 (1.4) | 1 (0.5) | 4 (1.7) | 2 (1.0) | 5 (2.8) |
| Lung cancer | 0 (0) | 1 (0.5) | 0 (0) | 0 (0) | 0 (0) |
| Skin cancer | 0 (0) | 5 (2.7) | 6 (2.6) | 6 (3.0) | 5 (2.8) |
| Blood cancer/lymphoma | 0 (0) | 1 (0.5) | 1 (0.4) | 0 (0) | 2 (1.1) |
| Acute or chronic leukaemia | 0 (0) | 1 (0.5) | 1 (0.4) | 1 (0.5) | 0 (0) |
| Cancer within the past five years that has metastasised | 6 (4.1) | 7 (3.7) | 14 (5.9) | 11 (5.5) | 4 (2.2) |
| Mild liver disease | 1 (0.7) | 1 (0.5) | 0 (0) | 1 (0.5) | 1 (0.6) |
| Hepatitis B | 1 (0.7) | 0 (0) | 0 (0) | 0 (0) | 0 (0) |
| Hepatitis C | 0 (0) | 0 (0) | 0 (0) | 0 (0) | 0 (0) |
| Cirrhosis | 0 (0) | 1 (0.5) | 0 (0) | 0 (0) | 0 (0) |
| Liver disease (moderate to severe) | 0 (0) | 1 (0.5) | 1 (0.4) | 3 (1.5) | 1 (0.6) |
| Chronic jaundice | 0 (0) | 1 (0.5) | 0 (0) | 1 (0.5) | 0 (0) |
| Liver failure | 0 (0) | 0 (0) | 0 (0) | 1 (0.5) | 0 (0) |
| Liver transplant | 0 (0) | 0 (0) | 0 (0) | 0 (0) | 0 (0) |
| AIDS or HIV | 0 (0) | 0 (0) | 0 (0) | 0 (0) | 0 (0) |
| Taking warfarin | 4 (2.7) | 8 (4.2) | 15 (6.4) | 17 (8.4) | 45 (24.7) |

**Supplementary Table 2.** Number and percentage of men and women with each health condition at baseline.

|  | Men  (N=311) | Women  (N=643) |
| --- | --- | --- |
| **Health conditions** | Yes, N (%) |  |
| Myocardial infarction (history of heart attacks) | 33 (10.5) | 20 (3.1) |
| Congestive heart failure | 2 (0.6) | 8 (1.2) |
| Hypertension/high blood pressure | 118 (37.7) | 241 (37.5) |
| Diagnosed depression | 7 (2.2) | 70 (10.9) |
| Peripheral vascular disease | 17 (5.4) | 30 (4.7) |
| Aortic aneurysm | 4 (1.3) | 3 (0.5) |
| Poor circulation | 8 (2.6) | 10 (3.0) |
| Cerebrovascular disease | 19 (6.1) | 31 (4.8) |
| Stroke | 8 (2.6) | 10 (1.6) |
| Cerebrovascular accident | 1 (0.3) | 0 (0) |
| Transient Ischemic attack | 6 (1.9) | 19 (3.0) |
| Dementia | 16 (5.1) | 33 (5.1) |
| Chronic bad chest | 29 (9.3) | 74 (11.5) |
| Asthma | 16 (5.1) | 49 (7.6) |
| Chronic obstructive pulmonary disease | 7 (2.3) | 21 (3.3) |
| Chronic bronchitis | 2 (0.6) | 6 (0.9) |
| Emphysema | 1 (0.3) | 3 (0.5) |
| Inflammation affecting the joints | 59 (18.9) | 187 (29.1) |
| Lupus | 1 (0.3) | 2 (0.3) |
| Rheumatoid arthritis | 33 (10.6) | 58 (9.0) |
| Connective tissue disease | 0 (0) | 5 (0.8) |
| Vasculitis | 0 (0) | 3 (0.5) |
| Peptic/stomach ulcer disease | 8 (2.6) | 19 (3.0) |
| Skin ulcer | 1 (0.3) | 8 (1.2) |
| Bed sores | 0 (0) | 0 (0) |
| Repeated cellulitis | 1 (0.3) | 6 (0.9) |
| Diabetes controlled with insulin or equivalent | 31 (9.9) | 44 (6.8) |
| Diabetes with end organ damage | 13 (4.2) | 19 (3.0) |
| Damage to the retina | 3 (1.0) | 6 (0.9) |
| Nerve damage | 0 (0) | 4 (0.6) |
| Kidney damage | 2 (0.6) | 3 (0.5) |
| Brittle diabetes | 0 (0) | 0 (0) |
| Moderate or severe chronic kidney disease | 7 (2.2) | 8 (1.2) |
| Hemiplegia | 1 (0.3) | 0 (0) |
| Cancer within the last five years | 47 (15.0) | 65 (10.1) |
| Breast cancer | 2 (0.6) | 12 (1.9) |
| Colon cancer | 2 (0.6) | 3 (0.5) |
| Prostate cancer | 14 (4.5) | 0 (0) |
| Lung cancer | 0 (0) | 1 (0.2) |
| Skin cancer | 14 (4.5) | 8 (1.3) |
| Blood cancer/lymphoma | 3 (1.0) | 1 (0.2) |
| Acute or chronic leukaemia | 1 (0.3) | 2 (0.3) |
| Cancer within the past five years that has metastasised | 10 (3.2) | 32 (5.0) |
| Mild liver disease | 1 (0.3) | 3 (0.5) |
| Hepatitis B | 0 (0) | 1 (0.2) |
| Hepatitis C | 0 (0) | 0 (0) |
| Cirrhosis | 0 (0) | 1 (0.2) |
| Liver disease (moderate to severe) | 1 (0.3) | 5 (0.8) |
| Chronic jaundice | 0 (0) | 2 (0.3) |
| Liver failure | 0 (0) | 1 (0.2) |
| Liver transplant | 0 (0) | 0 (0) |
| AIDS or HIV | 0 (0) | 0 (0) |
| Taking warfarin | 36 (11.4) | 33 (4.1) |

**Supplementary Table 3.** Number and percentage of participants with each health condition at baseline according to educational achievement.

|  | No qualifications  (N=232) | School leaving certificate age 16  (N=223) | School leaving certificate age 18  (N=272) | University level education  (N=224) |
| --- | --- | --- | --- | --- |
| **Health conditions** | Yes, N (%) |  |  |  |
| Myocardial infarction (history of heart attacks) | 15 (6.5) | 15 (6.7) | 17 (6.2) | 6 (2.7) |
| Congestive heart failure | 4 (1.7) | 0 (0) | 3 (1.1) | 3 (1.3) |
| Hypertension/high blood pressure | 99 (42.7) | 84 (37.7) | 98 (35.8) | 77 (34.2) |
| Diagnosed depression | 19 (8.2) | 18 (8.1) | 21 (7.7) | 18 (8.0) |
| Peripheral vascular disease | 15 (6.5) | 8 (3.6) | 15 (5.5) | 8 (3.6) |
| Aortic aneurysm | 3 (1.3) | 1 (0.5) | 3 (1.1) | 0 (0) |
| Poor circulation | 9 (3.9) | 6 (2.7) | 4 (1.5) | 7 (3.1) |
| Cerebrovascular disease | 9 (3.9) | 16 (7.2) | 13 (4.7) | 12 (5.3) |
| Stroke | 3 (1.3) | 8 (3.6) | 3 (1.1) | 4 (1.8) |
| Cerebrovascular accident | 0 (0) | 0 (0) | 1 (0.4) | 0 (0) |
| Transient Ischemic attack | 5 (2.2) | 8 (3.6) | 7 (2.6) | 5 (2.2) |
| Dementia | 16 (6.9) | 12 (5.4) | 11 (4.0) | 8 (3.6) |
| Chronic bad chest | 29 (12.5) | 27 (12.1) | 27 (9.9) | 20 (8.9) |
| Asthma | 20 (8.7) | 16 (7.2) | 14 (5.1) | 15 (6.7) |
| Chronic obstructive pulmonary disease | 7 (3.0) | 7 (3.1) | 10 (3.7) | 4 (1.8) |
| Chronic bronchitis | 5 (2.2) | 0 (0) | 2 (0.7) | 1 (0.5) |
| Emphysema | 1 (0.4) | 1 (0.5) | 1 (0.4) | 1 (0.5) |
| Inflammation affecting the joints | 75 (32.3) | 63 (28.3) | 69 (25.2) | 39 (17.3) |
| Lupus | 1 (0.4) | 1 (0.5) | 1 (0.4) | 0 (0) |
| Rheumatoid arthritis | 36 (15.6) | 22 (9.9) | 27 (9.9) | 6 (2.7) |
| Connective tissue disease | 1 (0.4) | 2 (0.9) | 0 (0) | 2 (0.9) |
| Vasculitis | 3 (1.3) | 0 (0) | 0 (0) | 0 (0) |
| Peptic/stomach ulcer disease | 8 (3.5) | 4 (1.8) | 12 (4.4) | 2 (0.9) |
| Skin ulcer | 2 (0.9) | 3 (1.4) | 2 (0.7) | 1 (0.5) |
| Bed sores | 0 (0) | 0 (0) | 0 (0) | 0 (0) |
| Repeated cellulitis | 1 (0.4) | 2 (0.9) | 2 (0.7) | 1 (0.5) |
| Diabetes controlled with insulin or equivalent | 23 (9.9) | 23 (10.3) | 20 (7.3) | 8 (3.6) |
| Diabetes with end organ damage | 9 (3.9) | 8 (3.6) | 10 (3.7) | 4 (1.8) |
| Damage to the retina | 2 (0.9) | 3 (1.4) | 3 (1.1) | 0 (0) |
| Nerve damage | 2 (0.9) | 2 (0.9) | 0 (0) | 0 (0) |
| Kidney damage | 1 (0.4) | 1 (0.5) | 2 (0.7) | 0 (0) |
| Brittle diabetes | 0 (0) | 0 (0) | 0 (0) | 0 (0) |
| Moderate or severe chronic kidney disease | 2 (1.3) | 2 (0.9) | 5 (1.8) | 4 (1.8) |
| Hemiplegia | 0 (0) | 1 (0.5) | 0 (0) | 0 (0) |
| Cancer within the last five years | 28 (12.1) | 21 (9.4) | 32 (11.7) | 31 (13.8) |
| Breast cancer | 1 (0.4) | 3 (1.4) | 2 (0.7) | 8 (3.6) |
| Colon cancer | 2 (0.9) | 1 (0.5) | 1 (0.4) | 1 (0.5) |
| Prostate cancer | 2 (0.9) | 0 (0) | 8 (2.9) | 4 (1.8) |
| Lung cancer | 0 (0) | 0 (0) | 0 (0) | 1 (0.5) |
| Skin cancer | 7 (3.0) | 2 (0.9) | 6 (2.2) | 7 (3.1) |
| Blood cancer/lymphoma | 2 (0.9) | 0 (0) | 0 (0) | 2 (0.9) |
| Acute or chronic leukaemia | 1 (0.4) | 0 (0) | 2 (0.7) | 0 (0) |
| Cancer within the past five years that has metastasised | 11 (4.7) | 10 (4.5) | 12 (4.4) | 9 (4.0) |
| Mild liver disease | 1 (0.4) | 1 (0.5) | 1 (0.4) | 1 (0.4) |
| Hepatitis B | 0 (0) | 1 (0.5) | 0 (0) | 0 (0) |
| Hepatitis C | 0 (0) | 0 (0) | 0 (0) | 0 (0) |
| Cirrhosis | 0 (0) | 0 (0) | 0 (0) | 1 (0) |
| Liver disease (moderate to severe) | 1 (0.4) | 2 (0.9) | 2 (0.7) | 1 (0.4) |
| Chronic jaundice | 1 (0.4) | 1 (0.5) | 0 (0) | 0 (0) |
| Liver failure | 0 (0) | 0 (0) | 1 (0.4) | 0 (0) |
| Liver transplant | 0 (0) | 0 (0) | 0 (0) | 0 (0) |
| AIDS or HIV | 0 (0) | 0 (0) | 0 (0) | 0 (0) |
| Taking warfarin | 15 (6.4) | 17 (7.6) | 24 (8.7) | 13 (5.8) |

**Supplementary Table 4.** Number and percentage of participants with each health condition at baseline according to hours of care per day.

|  | Less than one hour of care per day  (N=195) | One to ten hours of care per day (N=361) | More than ten hours of care per day  (N=412) |
| --- | --- | --- | --- |
| **Health conditions** | Yes, N (%) |  |  |
| Myocardial infarction (history of heart attacks) | 10 (5.1) | 20 (5.5) | 23 (5.6) |
| Congestive heart failure | 1 (0.5) | 1 (0.3) | 8 (1.9) |
| Hypertension/high blood pressure | 53 (27.2) | 134 (37.1) | 170 (41.3) |
| Diagnosed depression | 6 (3.1) | 30 (8.3) | 41 (10.0) |
| Peripheral vascular disease | 9 (4.6) | 19 (5.3) | 18 (4.4) |
| Aortic aneurysm | 2 (1.0) | 3 (0.8) | 2 (0.5) |
| Poor circulation | 2 (1.0) | 13 (3.6) | 12 (2.9) |
| Cerebrovascular disease | 8 (4.1) | 20 (5.5) | 20 (4.9) |
| Stroke | 3 (1.5) | 9 (2.5) | 6 (1.5) |
| Cerebrovascular accident | 0 (0) | 1 (0.3) | 0 (0) |
| Transient Ischemic attack | 3 (1.5) | 8 (2.2) | 13 (3.2) |
| Dementia | 8 (4.1) | 17 (4.7) | 23 (5.6) |
| Chronic bad chest | 17 (8.7) | 40 (11.1) | 44 (10.7) |
| Asthma | 13 (6.7) | 23 (6.4) | 27 (6.6) |
| Chronic obstructive pulmonary disease | 2 (1.0) | 11 (3.0) | 14 (3.4) |
| Chronic bronchitis | 2 (1.0) | 2 (0.6) | 4 (1.0) |
| Emphysema | 1 (0.5) | 1 (0.3) | 2 (0.5) |
| Inflammation affecting the joints | 47 (24.1) | 78 (21.6) | 119 (28.9) |
| Lupus | 0 (0) | 1 (0.3) | 1 (0.2) |
| Rheumatoid arthritis | 19 (9.7) | 24 (6.6) | 48 (11.7) |
| Connective tissue disease | 2 (1.0) | 2 (0.6) | 1 (0.2) |
| Vasculitis | 1 (0.5) | 1 (0.3) | 1 (0.2) |
| Peptic/stomach ulcer disease | 2 (1.0) | 11 (3.1) | 14 (3.4) |
| Skin ulcer | 1 (0.5) | 5 (1.4) | 3 (0.7) |
| Bed sores | 0 (0) | 0 (0) | 0 (0) |
| Repeated cellulitis | 1 (0.5) | 3 (0.8) | 3 (0.7) |
| Diabetes controlled with insulin or equivalent | 13 (6.7) | 31 (8.6) | 30 (7.3) |
| Diabetes with end organ damage | 7 (3.6) | 8 (0.8) | 17 (4.1) |
| Damage to the retina | 2 (1.0) | 2 (0.6) | 5 (1.2) |
| Nerve damage | 1 (0.5) | 1 (0.3) | 2 (0.5) |
| Kidney damage | 2 (1.0) | 0 (0) | 3 (0.7) |
| Brittle diabetes | 0 (0) | 0 (0) | 0 (0) |
| Moderate or severe chronic kidney disease | 2 (1.0) | 6 (1.7) | 7 (1.7) |
| Hemiplegia | 0 (0) | 0 (0) | 1 (0.2) |
| Cancer within the last five years | 25 (12.8) | 46 (12.7) | 39 (9.5) |
| Breast cancer | 2 (1.0) | 7 (1.9) | 5 (1.2) |
| Colon cancer | 0 (0) | 3 (0.8) | 2 (0.5) |
| Prostate cancer | 4 (2.1) | 77 (21.3) | 3 (0.7) |
| Lung cancer | 0 (0) | 1 (0.3) | 0 (0) |
| Skin cancer | 9 (4.6) | 9 (2.5) | 4 (1.0) |
| Blood cancer/lymphoma | 1 (0.5) | 1 (0.3) | 2 (0.5) |
| Acute or chronic leukaemia | 0 (0) | 0 (0) | 3 (0.7) |
| Cancer within the past five years that has metastasised | 10 (5.1) | 12 (3.3) | 19 (4.6) |
| Mild liver disease | 1 (0.5) | 0 (0) | 3 (0.7) |
| Hepatitis B | 0 (0) | 0 (0) | 1 (0.2) |
| Hepatitis C | 0 (0) | 0 (0) | 0 (0.) |
| Cirrhosis | 0 (0) | 0 (0) | 1 (0.2) |
| Liver disease (moderate to severe) | 0 (0) | 1 (0.3) | 5 (1.2) |
| Chronic jaundice | 0 (0) | 0 (0) | 2 (0.5) |
| Liver failure | 0 (0) | 0 (0) | 1 (0.2) |
| Liver transplant | 0 (0) | 0 (0) | 0 (0) |
| AIDS or HIV | 0 (0) | 0 (0) | 0 (0) |
| Taking warfarin | 73 (37.4) | 27 (7.5) | 31 (7.5) |

**Supplementary Table 5.** Number and percentage of participants with each health condition at baseline according to time since diagnosis in the person with dementia.

|  | Less than one year  (N=479) | Between one and two years (N=298) | Three or more years (N=134) |
| --- | --- | --- | --- |
| **Health conditions** | Yes, N (%) |  |  |
| Myocardial infarction (history of heart attacks) | 27 (5.6) | 16 (5.4) | 8 (6.0) |
| Congestive heart failure | 5 (1.0) | 2 (0.7) | 2 (1.5) |
| Hypertension/high blood pressure | 173 (36.1) | 106 (35.6) | 55 (41.0) |
| Diagnosed depression | 26 (5.4) | 32 (10.7) | 13 (9.7) |
| Peripheral vascular disease | 20 (4.2) | 18 (6.0) | 2 (1.5) |
| Aortic aneurysm | 4 (0.8) | 2 (0.7) | 0 (0) |
| Poor circulation | 10 (2.1) | 12 (4.0) | 1 (0.7) |
| Cerebrovascular disease | 14 (2.9) | 18 (6.0) | 12 (9.0) |
| Stroke | 4 (0.8) | 9 (3.0) | 3 (2.2) |
| Cerebrovascular accident | 0 (0) | 0 (0) | 1 (0.7) |
| Transient Ischemic attack | 6 (1.3) | 9 (3.0) | 8 (6.0) |
| Dementia | 25 (5.2) | 17 (5.7) | 4 (3.0) |
| Chronic bad chest | 51 (10.6) | 25 (8.4) | 18 (13.4) |
| Asthma | 35 (7.3) | 15 (5.0) | 9 (6.7) |
| Chronic obstructive pulmonary disease | 11 (2.3) | 7 (2.3) | 8 (6.0) |
| Chronic bronchitis | 3 (0.6) | 1 (0.3) | 4 (3.0) |
| Emphysema | 0 (0) | 3 (1.0) | 1 (0.7) |
| Inflammation affecting the joints | 126 (26.3) | 71 (23.8) | 36 (26.9) |
| Lupus | 2 (0.4) | 0 (0) | 1 (0.7) |
| Rheumatoid arthritis | 46 (9.6) | 27 (9.1) | 11 (8.2) |
| Connective tissue disease | 1 (0.2) | 2 (0.7) | 1 (0.7) |
| Vasculitis | 0 (0) | 1 (0.3) | 2 (1.5) |
| Peptic/stomach ulcer disease | 11 (2.3) | 8 (2.7) | 7 (5.2) |
| Skin ulcer | 3 (0.6) | 4 (1.3) | 2 (1.5) |
| Bed sores | 0 (0) | 0 (0) | 0 (0) |
| Repeated cellulitis | 2 (0.4) | 3 (1.0) | 2 (1.5) |
| Diabetes controlled with insulin or equivalent | 43 (9.0) | 22 (7.4) | 5 (3.7) |
| Diabetes with end organ damage | 16 (3.3) | 11 (3.7) | 4 (3.0) |
| Damage to the retina | 5 (1.0) | 2 (0.7) | 2 (1.5) |
| Nerve damage | 2 (0.4) | 1 (0.3) | 1 (0.7) |
| Kidney damage | 2 (0.4) | 2 (0.7) | 0 (0) |
| Brittle diabetes | 0 (0) | 0 (0) | 0 (0) |
| Moderate or severe chronic kidney disease | 6 (1.3) | 8 (2.7) | 1 (0.7) |
| Hemiplegia | 0 (0) | 0 (0) | 1 (0.7) |
| Cancer within the last five years | 55 (11.5) | 38 (12.8) | 13 (9.7) |
| Breast cancer | 8 (1.7) | 6 (2.0) | 0 (0) |
| Colon cancer | 3 (0.6) | 2 (0.7) | 0 (0) |
| Prostate cancer | 6 (1.3) | 6 (2.0) | 1 (0.7) |
| Lung cancer | 0 (0) | 1 (0.3) | 0 (0) |
| Skin cancer | 11 (2.3) | 6 (2.0) | 3 (2.2) |
| Blood cancer/lymphoma | 3 (0.6) | 1 (0.3) | 0 (0) |
| Acute or chronic leukaemia | 0 (0) | 1 (0.3) | 1 (0.7) |
| Cancer within the past five years that has metastasised | 20 (4.2) | 15 (5.0) | 5 (3.7) |
| Mild liver disease | 2 (0.4) | 1 (0.3) | 1 (0.7) |
| Hepatitis B | 1 (0.2) | 0 (0.) | 0 (0) |
| Hepatitis C | 0 (0) | 0 (0) | 0 (0) |
| Cirrhosis | 0 (0) | 0 (0) | 1 (0.7) |
| Liver disease (moderate to severe) | 1 (0.2) | 2 (0.7) | 1 (0.7) |
| Chronic jaundice | 0 (0) | 2 (0.7) | 0 (0) |
| Liver failure | 0 (0) | 0 (0) | 1 (0.7) |
| Liver transplant | 0 (0) | 0 (0) | 0 (0) |
| AIDS or HIV | 0 (0) | 0 (0) | 0 (0) |
| Taking warfarin | 31 (6.5) | 91 (30.5) | 11 (8.2) |

**Supplementary Table 6: Time-varying covariates of number of health conditions.**

|  | Informant-rated functional ability  IRR (95% CI) | Neuropsychiatric symptoms  IRR (95% CI) | Cognition of the person with dementia  IRR (95% CI) | Multivariable model comprising informant-rated functional ability, neuropsychiatric symptoms, and cognition of the person with dementia  IRR (95% CI) |
| --- | --- | --- | --- | --- |
| Intercept | 1.95 (1.51; 2.51) | 1.56 (1.25; 1.94) | 1.45 (1.04; 2.03) | 1.36 (0.82; 2.26) |
| Time | 1.19 (1.13; 1.26) | 1.20 (1.14; 1.25) | 1.20 (1.14; 1.27) | 1.20 (1.13; 1.28) |
| Within-person effects: Informant-rated functional ability | 0.99 (0.88; 1.10) |  |  | 1.02 (0.90; 1.17) |
| Between-person effects: Informant-rated functional ability | 0.98 (0.90; 1.07) |  |  | 0.96 (0.87; 1.07) |
| Within-person effects: Neuropsychiatric symptoms |  | 0.99 (0.97; 1.02) |  | 1.00 (0.96; 1.03) |
| Between-person effects: Neuropsychiatric symptoms |  | 1.05 (1.02; 1.08) |  | 1.05 (1.02; 1.09) |
| Within-person effects: Cognition of the person with dementia |  |  | 1.01 (0.95; 1.07) | 1.01 (0.95; 1.08) |
| Between-person effects: Cognition of the person with dementia |  |  | 1.04 (0.99; 1.09) | 1.04 (0.99; 1.10) |

All adjusted for age, sex, education, hours of care per day (time-varying), and time since diagnosis. IRR= Incidence rate ratio.

**Supplementary Table 7: Number of health conditions and covariates as time-varying predictors of stress, positive aspects of caregiving, and social network.**

|  | Stress  (estimate, 95% CI) | Positive aspects of caregiving  (estimate, 95% CI) | Social network  (estimate, 95% CI) |
| --- | --- | --- | --- |
| Unadjusted model |  |  |  |
| Within-person effects | 2.49 (1.98; 3.00) | -0.19 (-0.59; 0.20) | -0.39 (-0.66; -0.13) |
| Between-person effects | 0.53 (0.15; 0.92) | 0.37 (0.08; 0.66) | -0.13 (-0.34; 0.08) |
| Adjusted model one |  |  |  |
| Within-person effects | 2.20 (1.69; 2.71) | -0.21 (-0.63; 0.20) | -0.39 (-0.67; -0.11) |
| Between-person effects | 0.43 (0.06; 0.80) | 0.20 (-0.10; 0.49) | -0.12 (-0.34; 0.10) |
| Adjusted model two |  |  |  |
| Within-person effects | 1.19 (0.69; 1.69) | -0.24 (-0.73; 0.25) | -0.32 (-0.64; 0.00) |
| Between-person effects | 0.14 (-0.18; 0.46) | 0.23 (-0.08; 0.53) | -0.00 (-0.23; 0.23) |

Adjusted model one: age, sex, education, hours of care per day, time since diagnosis. Adjusted model two: age, sex, education, hours of care per day, time since diagnosis, neuropsychiatric symptoms, informant-rated functional ability, and cognition of the person with dementia.
